# A novel mutation in *PTHLH* in a family with a variable phenotype with brachydactyly, short stature, oligodontia and developmental delay

**DOI:** 10.1016/j.bonr.2023.101699

**Published:** 2023-07-15

**Authors:** Mirjam E.A. Scheffer-Rath, Hermine E. Veenstra-Knol, Annemieke M. Boot

**Affiliations:** a Department of Pediatric Endocrinology, University Medical Center Groningen, University of Groningen, P.O. Box 30.001, 9700 RB Groningen, the Netherlands; b Department of Genetics, University Medical Center Groningen, University of Groningen, P.O. Box 30.001, 9700 RB Groningen, the Netherlands

**Keywords:** *PTHLH*, PTH-related peptide, Brachydactyly type E, Disproportionate short stature, Developmental delay

## Abstract

Mutations in *PTHLH* (PTH-like hormone), cause brachydactyly type E (BDE) characterized by shortening of metacarpals, metatarsals and/or phalanges with short stature. In this report we describe three siblings and their mother with a novel heterozygous mutation c.25 T > C, p.Trp9Arg in exon 2 of the *PTHLH* gene. Beside the known clinical features of PTHLH mutations all had a delay in speech and language development, unknown if this is related to the mutation. Patients with PTHLH mutation may have a variable phenotypic presentation.

## 1 Introduction

Brachydactyly type E (BDE) is a congenital malformation of the hands and feet comprising of shortening of metacarpals and metatarsals with frequently involvement of phalanges. BDE can be isolated, caused by a heterozygous *HOXD13* mutation (OMIM #113300) or microdeletions of 2q37, or part of a syndrome (Klopocki et al., 2010; Pereda et al., 2013). The syndromic forms of BDE can be associated with hormonal resistance and short stature such as in pseudohypoparathyroidism type 1A (PHP1A; OMIM #103580) or acrodysostosis (OMIM #101800 and #614613) or with short stature without hormonal resistance such as in pseudopseudohypoparathyroidism (PPHP; OMIM #612463), hypertension with brachydactyly syndrome (OMIM #112410), Turner syndrome or BDE caused by *PTHLH* mutations (OMIM #613382) (Pereda et al., 2013). *PTHLH* (PTH-like hormone) is the gene encoding PTH-related peptide (PTHrP). PTHrP is important in bone development which is demonstrated by PTHrP knock-out mice in which histological examination showed a reduction of chondrocyte proliferation, associated with premature maturation of chondrocytes and accelerated bone formation. These PTHrP knockout mice were stillborn with disproportionately short limbs, short snouts and mandibles and domed skulls (Karaplis et al., 1994). In addition to the action of PTHrP in bone development, it has biological actions in mammary gland, dental development, gestation, reproduction, lactation, smooth muscle relaxation, central nervous system activity, and effect on skin and hair follicles (de Papp and Stewart, 1993; Strewler, 2000). Here we report a family with a novel heterozygous mutation c.25 T > C, p.Trp9Arg in the *PTHLH* gene and a variable phenotype.

## 2 Clinical reports

### 2.1 Clinical presentation

#### 2.1.1 Patient 1

The boy, one of a twin, was born prematurely after 34 weeks and 2 days of gestation with a birth weight of 1740 g (−1.18 SD) and length of 40 cm (−2.22 SD). The pregnancy was complicated by preeclampsia from 34 weeks and Apgar score after 5 min after birth was 8. At the age of 3 months he was operated on an inguinal hernia. He presented at the age of 3 years with a disproportionate short stature (height 91.4 cm −2.3 SD), sitting height 56.7 cm (sitting height for height +3.4 SD), arm span 82 cm (arm span for height −0.67 SD). He also had frontal bossing with a depressed nasal bridge. His speech/language development was delayed without hearing loss and he was walking unassisted from the age of 18 months. There was no oligodontia. His twin sister is healthy with a normal height and development. His mother's height is 149.5 cm (−3.4 SD) (she is patient 2, see below), and his father's height is 179.9 cm (0.57 SD). The maternal grandparents have normal heights (165 cm and 182 cm). His bone age was around 2.5 year advanced to his chronological age and skeletal survey showed enhanced lumbar lordosis and no decrease in the lumbar interpedicular distances which can be observed in hypo- or achondroplasia. From the age of 5 years he had short metacarpal 4, known as BDE and short metatarsals. Laboratory results showed normal serum calcium, phosphate, alkaline phosphatase, PTH and immeasurable PTHrP (Table 1).

**Table 1:** Laboratory results of patient 1 and 2. Between blankets are the reference values.

	Calcium (2.2–2.6 mmol/l)	Phosphate (1.0–1.8 mmol/l)	Alkaline phosphatase (<425 U/l)	PTH (<7 pmol/l)	PTHrp (<2 pmol/l)	25 OH vit D (>50 nmol/l)
Pt 1 5.7 yrs	2.43	1.70	213	3.3	<0.3	59
Pt 3 2.3 yrs	2.46	1.31	201	1.6	1.3	99.4

**Fig. 1:**
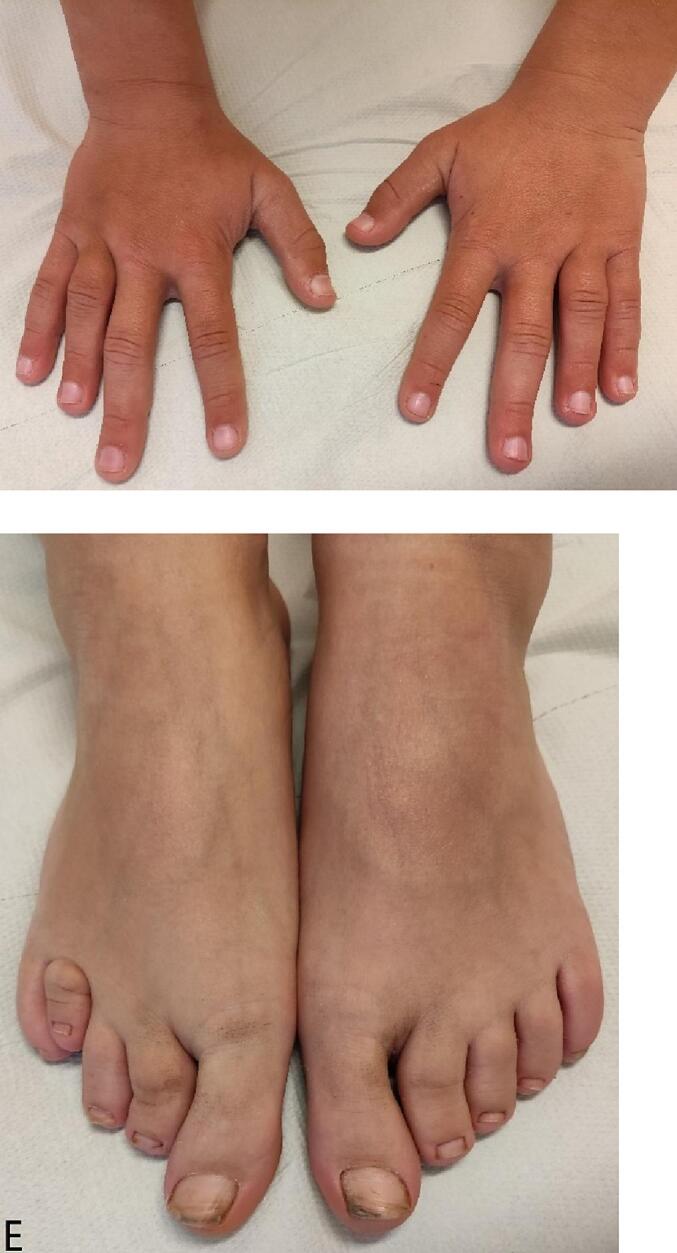
Hands and feet of patient 1.

**Fig. 2:**
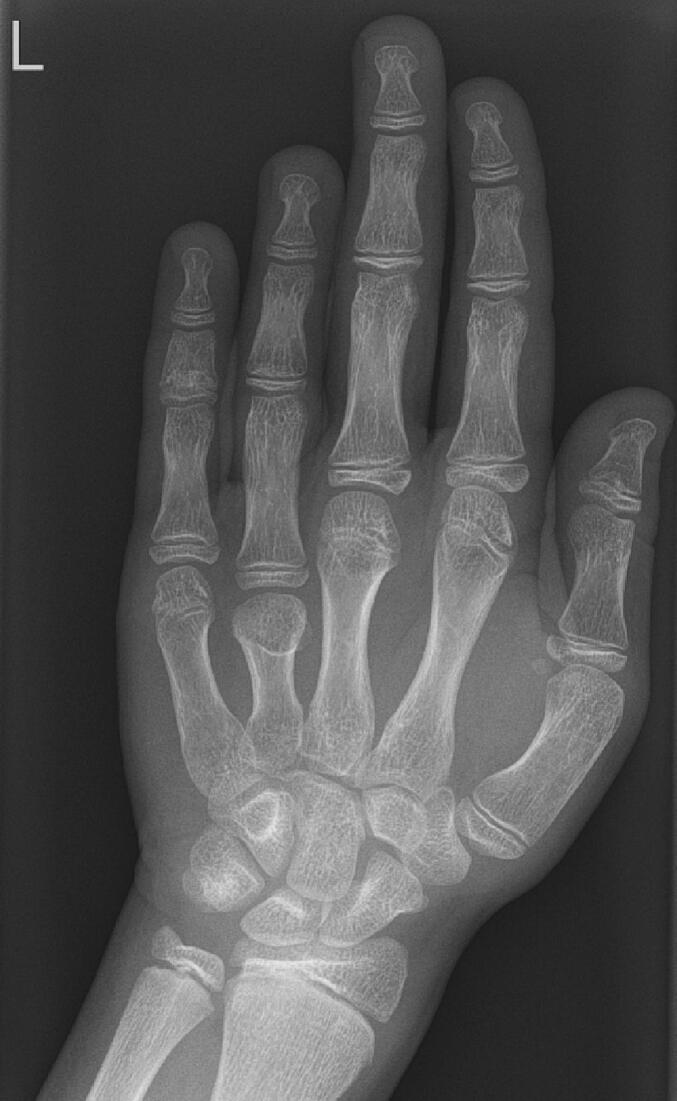
Hand X-ray of patient 1 at the age 12 years.

**Fig. 3:**
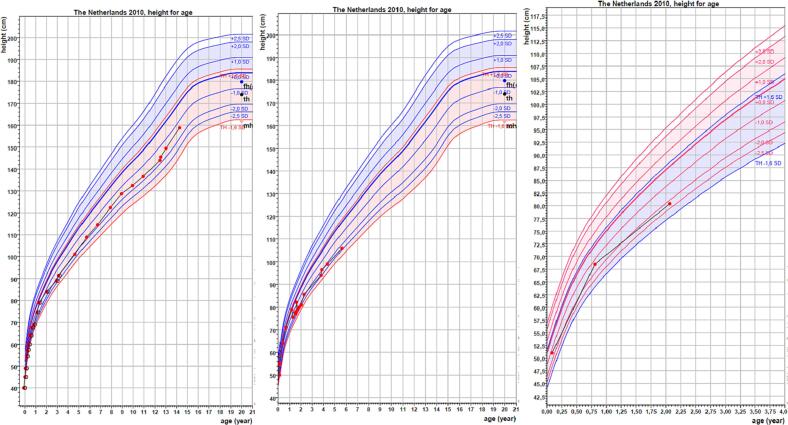
Growth charts of patients 1, 3 and 4. Target height range is in red in boys and in blue in the girl.

At the age of 7.8 years a non-verbal intelligent test was performed. The non-verbal IQ score of our patient was 79. He attends a school for children with learning disabilities. His height at the age of 13 years is 149.4 cm (−1.52 SD), weight 40.2 kg, weight/height +0.73 SD, sitting height 84.3 cm, sitting height/height ratio 0.56 (+5.36 SD) and Tanner stage G4P3A2 with testicular volume of 12–15 ml. His hands and feet are shown in Fig. 1 and an X-ray of his left hand is presented in Fig. 2. His growth curve is shown in Fig. 3.

#### 2.1.2 Patient 2

The mother of patient 1 is 149.5 cm (−3.4 SD) tall, her sitting height is 82.7 cm (sitting height for height + 2 SD) and also has BDE with a short metacarpal 4 of the right hand and short metatarsal 4 of the left foot. She was born term without any problems after birth. She also had a history of speech development delay (speech therapy in first en second grade of primary school) and her highest education is preparatory vocational education care profile. She is not working anymore because of back and fatigue complaints. She has no oligodontia. She had normal breast development but was not able to breastfeed her children. She had fertility treatment on all her children. One spontaneous pregnancy ended in miscarriage.

#### 2.1.3 Patient 3

**Fig. 4:**
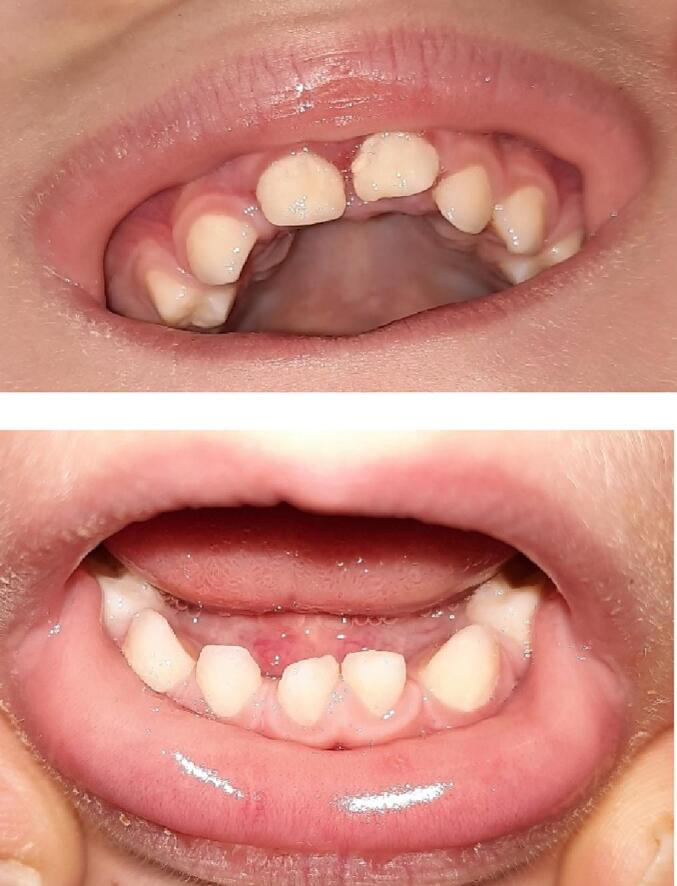
Upper and lower jaw of patient 3 with oligodontia.

The 8 years younger brother of patient 1, was born after 38 weeks and 5 days of gestation with a birth weight of 2805 g (−1.24 SD), length of 45 cm (−2.53 SD) and Apgar score of 10 after 5 min. When he was almost 2 years old a tonsillectomy was performed because of pediatric sleep apnea syndrome. He was walking unassisted at the age of 16 months. At the age of 2.3 years he had a speech and language development delay, feeding problem (only eating mashed food) and disproportionate short stature with a height of 85.5 cm (−1.71 SD) and sitting height 53.7 cm (sitting height for height + 1.98 SD). He misses 1 upper and 1 lower lateral incisor (Fig. 4). Laboratory results are shown in Table 1 and growth curve in Fig. 3.

At the age of 4 years he started regular primary education (without speech therapy) and he had no more feeding problems.

#### 2.1.4 Patient 4

The younger sister of patient 1 and 3 presented at the age of 9 months with a disproportionate short stature with a height of 68.5 cm (−1.35 SD), sitting height of 46.8 cm (sitting height for height + 1.16 SD). She was born term with a birth weight of 2865 g (−1 SD) and Apgar score of 9 after 5 min. At the age of two years she did not speak yet. She only ate mashed food. Her growth curve is shown in Fig. 3.

Written informed consent was obtained from both parents for publication of this case report.

### 2.2 Genetic investigations

To evaluate the cause of the disproportional short stature chromosome microarray analysis was performed in patient 1 and 2 which showed no clinical significant abnormalities. Thereafter direct nucleotide sequence analysis and MLPA of *FGFR3*, *SHOX* and *COL2A1* in patient 1 revealed no pathogenic variants and in patient 2 *GNAS* analysis showed no abnormalities.

**Fig. 5:**
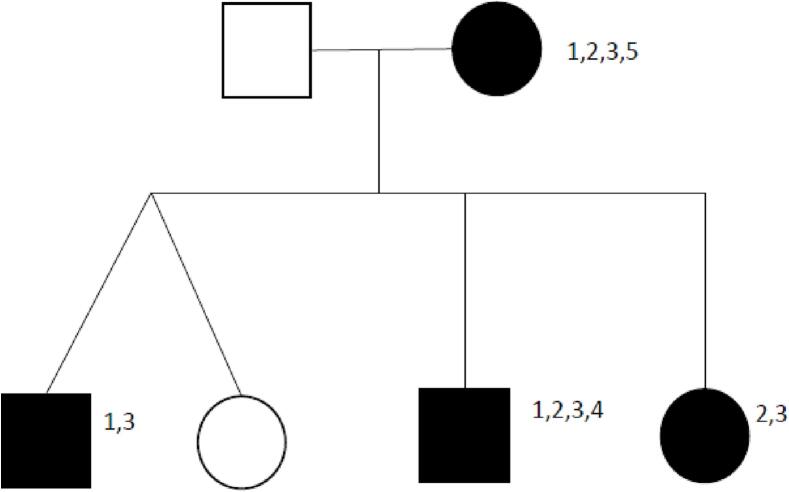
1: Brachydactyly; 2: height < −2 SD; 3: speech development delay; 4: dental findings; 5: problems with breast feeding. Black symbols mean PTHLH mutation present.

Because of BDE and short stature, sequencing of *PTHLH* was performed in the mother and revealed a heterozygous, missense mutation in exon 2 of *PTHLH* gene (c.25 T > C, p.Trp9Arg). Thereafter, this mutation was also found in her sons and youngest daughter (patient 1, 3 and 4, Fig. 5). This variant was not previously reported. The mutation causes a change of amino acid in the PTHLH protein. In silico prediction supports a deleterious effect on the gene, aggregated score 0.863 (moderate supporting).

## 3 Discussion

We describe four patients of one family with a novel mutation in exon 2 of the *PTHLH* gene. The first patient presented with developmental delay and disproportionate short stature with skeletal abnormalities. His mother also had a short stature with BDE and history of speech developmental delay. Genetic analysis revealed a mutation in *PTHLH* gene which was also present in her two sons and later in her daughter.

PTHrP, which is coded by *PTHLH*, binds to the PTH/PTHrP-receptor (PTHR1) and is essential for normal cartilage/bone development. No functional studies have been done in our family. The amino-acid substitution at codon 9 could have effect on the signal peptide cleavage site, resulting in PTHLH loss of function (Wang et al., 2015). This can lead to increased chondrocyte apoptosis and premature closure of growth plates resulting in BDE, advanced bone age and short stature like in our patients (Reyes et al., 2020; Reyes et al., 2019). PTHR1 activates the Gsα-cAMP-PKA-PDE4D signaling pathway and mutations in other genes encoding for proteins in this pathway, namely *GNAS* (leading to PHP or PPHP), *PRKAR1A* and *PDE4D* (leading to acrodysostosis type 1 and 2 respectively), can also lead to BDE and short stature (Reyes et al., 2019; Thomas-Teinturier et al., 2016). Neurocognitive impairment may occur in patients with PHP1A and acrodysostosis type 2 (Mantovani et al., 2018).

To date, twelve *PTHLH* mutations (Bae et al., 2018; Elli et al., 2022; Fu et al., 2019; Jamsheer et al., 2016; Klopocki et al., 2010; Pereda et al., 2017; Reyes et al., 2019; Thomas-Teinturier et al., 2016; Wang et al., 2015), two families with *PTHLH* deletions (Huang et al., 2019; Klopocki et al., 2010) and two families with balanced translocations leading to repression of *PTHLH* (Maass et al., 2012; Maass et al., 2010) causing BDE have been reported to our knowledge (see Table 2). In 2010, Klopocki et al. reported the first 5 unrelated families with gene defects in *PTHLH*. All affected individuals presented with BDE and 6 of 8 affected individuals presented with short stature (height between −2 and −2.8 SD). In 4 out of 5 families point mutations were observed and in one family a microdeletion on chromosome 12p was detected affecting 6 genes including *PTHLH* (Klopocki et al., 2010).The family with this microdeletion presented with learning difficulties in addition to BDE and short stature. Since this family was the only family with learning difficulties, the authors stated that the deletion of the 5 genes distal to *PTHLH* most likely accounted for this. In our family with a point mutation in *PTHLH* gene all four patients have (a history of) speech development delay. Our index patient with persisting learning disabilities, was born prematurely after 34 weeks gestation which may also affected his speech and language development (Guarini et al., 2009). However, his twin sister who is lacking the *PTHLH* mutation, does not have learning disabilities. In addition, his mother, younger brother and sister also have (a history of) speech development delay and were not born prematurely. To our knowledge, no other reports on learning disabilities in patients with *PTHLH* mutations have been published to date.

**Table 2:** Point mutations and deletions in PTHLH gene and clinical features.

Mutation	Protein	Probable location	BDE	Short stature	Teeth problems	Abnormal breast development	Reference
c.2 T > C	p.(M1?)	Signal peptide	Y	Y	NI	NI	Elli et al., 2022
c.25 T > C	p.(W9R)	Signal peptide	Y	Y	Oligodontia in some	No breast feeding possible	This report
c.44 T > G	p.(L15R)	Signal peptide	Y	Y	N	NI	Wang et al., 2015
c.47_101del	NMD		Y	Y	Dental malpositions	Small breasts	Thomas-Teinturier et al., 2016
c.101del	NMD		Y	N	N	N	Thomas-Teinturier et al., 2016
c.125A > C	p.(Q42P)	PTHrp	Y	NI	NI	NI	Fu et al., 2019
c.131 T > C	p.(L44P)	PTHrp	Y	N	Problems with tooth eruption	NI	Klopocki et al., 2010
c.166C > T	p.(R56X)	PTHrp	Y	Y/N	Delayed eruption of definitive molar teeth	NI	Jamsheer et al., 2016; Pereda et al., 2017; Elli et al., 2022
c.169C > T	p.(R57X)	PTHrp	Y	N	N	N	Bae et al., 2018
c.179 T > C	p.(L60P)	PTHrp	Y	Y	Problems with tooth eruption	NI	Klopocki et al., 2010
c.258del	p.(N87X)	PTHrp	Y	N	NI	NI	Jamsheer et al., 2016
c.299del	p.(E100X)	PTHrp	Y	Y	Supernumerary tooth	NI	Elli et al., 2022
c.358A > T	p.(K120X)	PTHrp	Y	Y	Oligodontia	NI	Klopocki et al., 2010
c.532A > G	p.(X178W)	PTHrp	Y	Y	NI	NI	Klopocki et al., 2010

Legends; N=Not present, Y = Yes present, NI=No Information, NMD = Nonsense-Mediated Decay, PTHrp = PTH related protein, Probable location of protein (Wang et al., 2015).

In 2015 Thomas-Teinturier et al. reported on 2 novel *PTHLH* mutations and reviewed previously reported patients with *PTHLH* mutations or deletions and found that all patients had BDE with intrafamilial variability in regard to number of digits affected, 19 out of 27 patients had short stature (below −2 SD or in the lower range of normal) and 3 out of 27 had oligodontia/problems with tooth eruption. They were the first to report on a patient without breast development at the age of 12.8 years despite a uterine length of 54 mm and serum estradiol level of 162 pmol/l. It must be mentioned that not all patients with PTHLH mutations had reached their final height at the time of publication, therefore it could be that some patients without a short stature will have a short final height due to an advanced bone age. However, there are adults reported with *PTHLH* mutations without a short stature suggesting a variable penetrance of the gene defect (Bae et al., 2018; Jamsheer et al., 2016).

After the review of Thomas-Teinturier et al., another 7 clinical reports on BDE caused by *PTHLH* mutations (Bae et al., 2018; Fu et al., 2019; Jamsheer et al., 2016; Pereda et al., 2018; Pereda et al., 2017; Elli et al., 2022) or deletions (Huang et al., 2019) were published. Jamsheer et al. (2016) firstly reported on a patient with a *PTHLH* mutation causing short stature and delayed bone age, however this girl also had hypothyroidism with elevated anti-TPO antibodies which can lead to a delay in bone age. Pereda et al. (2017) compared the published X-rays with the Greulich and Pyle atlas and concluded that the bone age of the patients in the report of Jamsheer and colleagues was also advanced. Huang et al. (2019) reported on a family with a 3.06-Mb deletion (chr12:25473650-28536747) affecting 23 genes including *PTHLH*. This family presented with short stature, BDE and pectus carinatum, of which the latter has not been previously reported to be caused by *PTHLH* mutation/deletions. To our knowledge, the other publications did not report any new clinical characteristics beyond the already known inter- and intrafamilial variable BDE, short stature, short arm span, advanced bone age, dental anomalies, macrocephaly, frontal bossing, depressed nasal root and learning disabilities.

## 4 Conclusion

Based on this clinical report and reviewing the literature there is no clear genotype-phenotype correlation in *PTHLH* mutations related to BDE, short stature, dental anomalies and other clinical characteristics. This is the second report on learning disabilities in *PTHLH* defects and first report of a *PTHLH* point mutation in a family with speech and/or language development delay.

## Funding

This research did not receive any specific grant from funding agencies in the public, commercial, or not-for-profit sectors.

## CRediT authorship contribution statement

**Mirjam E.A. Scheffer-Rath:** Writing – original draft, Investigation, Data curation. **Hermine E. Veenstra-Knol:** Writing – review & editing, Conceptualization. **Annemieke M. Boot:** Writing – review & editing, Supervision, Data curation, Conceptualization.

## Declaration of competing interest

The authors declare that they have no known competing financial interests or personal relationships that could have appeared to influence the work reported in this paper.

## Data Availability

Data will be made available on request.
